# Possible role of SIRT1 and SIRT3 in post-translational modifications in human breast milk during the neonatal period

**DOI:** 10.1007/s00726-022-03197-7

**Published:** 2022-08-17

**Authors:** Svetlana Baskal, Bibiana Beckmann, Laura Stahmer, Corinna Peter, Bettina Bohnhorst, Anibh Martin Das, Dimitrios Tsikas

**Affiliations:** 1 Institute of Toxicology, Core Unit Proteomics, Hannover Medical School, Carl-Neuberg-Strasse 1, 30625 Hannover, Germany; 2 Clinic for Pediatric Kidney, Liver and Metabolic Diseases, Hannover Medical School, 30623 Hannover, Germany; 3 Clinic for Pediatric Pneumology, Allergology and Neonatology, Hannover Medical School, 30623 Hannover, Germany

**Keywords:** AGEs, Amino acids, Asymmetric dimethylarginine, Citrullination, Human breast milk, Oxidative stress, PTM, Sirtuins

## Abstract

We measured free and proteinic concentrations of native and modified amino acids from post-translational modifications (PTMs) and correlated them with the activity of SIRT1 and SIRT3 in the pellet and aqueous phases of human breast milk samples of ten lactating women during the neonatal period. SIRT1 and SIRT3 correlated directly with citrullination, asymmetric dimethylation and glycation of L-arginine, hydroxylation and glycation of L-lysine. SIRT1 and SIRT3 correlated inversely with the hydroxylation of L-proline. SIRT1 and SITR3 tended to correlate inversely with oxidative stress measured as malondialdehyde. Our study suggests that SIRT1 and SIRT3 may modulate PTMs in human breast milk cells.

## Introduction

Protein *N*-acetylation is one of the most frequent post-translational modifications (PTMs) (Tsikas 2022) and plays a central role in regulating protein function. The sirtuins (silent information regulators, SIRTs) class III histone deacetylases (HDACs) catalyze the deacetylation of *N*-acetylated proteins. SIRTs are involved in various physiological and patho-physiological processes, such as aging, mitochondrial biogenesis, regulation of metabolism, oxidative stress, apoptosis, and inflammation. SIRTs consist of seven proteins (SIRT1–7) that are located in different subcellular compartments. SIRT1 can be found in the nucleus and cytoplasm, SIRT3 is in the mitochondria. The complex network of SIRT1 and SIRT3 involves different cellular compartments, transcriptional activation, PTMs and a plethora of secondary effectors. The mode of interaction between SIRT1 and SIRT3 is considered as a prominent case of molecular ‘job-sharing’ (Brennmoehl and Hoeflich 2013). SIRT1 and SIRT3 control biogenesis, energy, lipid and glucose metabolism (Brennmoehl and Hoeflich 2013; Nogueiras et al. 2012; Das and Dabke 2021; Tabrizi et al. 2022).

Nutrition may impact SIRT1 and SIRT3 in human adults (Potthast et al. 2020). Sirtuin activity in breast milk may have a beneficial impact on the health of neonates and beyond. Sirtuins were suggested to play a role in pre- and post-natal programming, which may result in (metabolic) diseases during later life (Maissan et al. 2021). Little is known about sirtuins in human breast milk. Perinatal factors may have impact on the amount of SIRT3 in early human breast milk (Nyárády et al. 2020).

Recently, we found that active SIRT1 and SIRT3 are present both in the cellular pellet and in the aqueous phase of centrifuged human breast milk (Hase et al. 2021). In the present study, we measured the concentration of free and proteinic native and modified amino acids from PTMs and investigated their correlations with the activity of SIRT1 and SIRT3 in the pellet and the aqueous phase of human breast milk samples of ten lactating women during the neonatal period. We found many correlations of SIRT1 and SIRT3 activity with native and modified amino acids, suggesting possible roles of SIRT1 and SIRT3 in the modulation of PTMs and human breast milk.

## Materials and methods

### Lactation study

This study was approved by the ethical board of Hannover Medical School (EC No 8482_BO_K_2019), and written informed consent from all mothers was obtained before participation. Eight mothers (mean age, 34 years) delivered preterm, two mothers (mean age, 33 years) delivered at term. All mothers considered healthy; none of them suffered from metabolic diseases (diabetes mellitus) or metabolic pregnancy complications (e.g., pre-eclampsia, HELLP syndrome). Mothers were mostly non-smoking with an omnivore diet, and the included newborns were mainly hospitalized for prematurity.

The breast milk was mechanically pumped randomly during the day and stored at − 80 °C until analysis. Sample collection and analysis of sirtuins including the characterization of donating mothers is described elsewhere in detail (Hase et al. 2021). In the present study, we analyzed 35 breast milk samples from 10 mothers. Nine samples were obtained on days 7 and 21, 10 on day 14, and 7 samples on day 28 of lactation. Breast milk samples were centrifuged (4 °C, 8875 × g, 30 min) and separated into the cell pellet (pt), the aqueous phase (aq), and a lipid layer (Witkowska-Zimny and Kaminska-El-Hassan 2017). SIRT1 and SIRT3 proteins and their activity were measured only in the aqueous phase (SIRT1aq and SIRT3aq) and in the cell pellet (SIRT1pt and SIRT3pt). Amino acids, nitrite, nitrate, creatinine and malondialdehyde were measured in non-centrifuged milk samples as described below.

### Measurement of amino acids and other biomolecules in breast milk by stable-isotope dilution gas chromatography-mass spectrometry (GC–MS)

Breast milk samples were thawed slowly in an ice-bath to minimize changes in milk composition. Thawed milk samples were vortexed, proportioned and subjected to analysis. Free and total amino acids including free amino acid metabolites of PTMs were measured in parallel each in 10-µL aliquots of milk as reported elsewhere for human serum, plasma and urine (Bollenbach et al. 2018, 2019; Baskal et al. 2022). Free amino acids were measured after incubation of 10-µL aliquots of milk samples at room temperature for 20 h. Total amino acids were measured after incubation of additional 10-µL aliquots of milk samples at 110 °C for 20 h for hydrolysis in 6 M HCl. Proteinic (p) amino acids were calculated by subtracting free from of total amino acids. Citrullination (%) was calculated by dividing proteinic citrulline (pCit) by proteinic arginine (pArg) concentration and by multiplying the outcome by 100. Dimethylamine (DMA), the major metabolite of asymmetric dimethylarginine (ADMA) (Tsikas 2020), was measured in 10-µL aliquots of the centrifuged milk samples as reported elsewhere for human plasma and serum DMA (Chobanyan et al. 2007). Nitrite, nitrate, creatinine and malondialdehyde (MDA), a biomarker of oxidative stress (Tsikas 2017), were measured simultaneously in 120-µL aliquots of breast milk as described previously (Tsikas 2000). The equilibrium constant of the formation of the non-proteinogenic homoarginine (hArg), i.e., *K*_harg_, was calculated as described elsewhere (Tsikas and Redfors 2022) using the concentrations of hArg, ornithine (Orn), arginine (Arg) and lysine (Lys) measured in the milk samples. Analyses were performed on a GC–MS device consisting of a single quadrupole mass spectrometer model ISQ, a Trace 1210 series gas chromatograph and an AS1310 autosampler (all from ThermoFisher; Dreieich, Germany).

### Statistical analyses and data presentation

Data analysis and preparation of Figures were performed using GraphPad Prism 7 for Windows (GraphPad Software, San Diego, CA, USA). Wilcoxon matched-pairs signed rank test and Spearman correlations were performed. A two-sided *P* value of less than 0.05 was considered statistically significant. Concentrations are presented as median with interquartile range (IQR) or as mean with standard deviation (SD) or with error of the mean (SEM) as appropriate.

## Results

The concentrations of the analytes measured in the human breast milk samples of the present study are summarized in Table 1. The concentrations of all analytes are comparable to those measured in plasma and serum samples of healthy humans measured by us and others. The protein concentration (in g/L) was 11 ± 0.5 on day 7, 9.2 ± 0.4 on day 14, 7.6 ± 0.4 on day 21, and 7.8 ± 0.4 on day 28.

**Table 1:** Concentrations of free (mean ± SD, µM) and total (mean ± SD, mM or µM) amino acids (AA), and various other metabolites (median [IQR]) measured in the human breast milk samples of ten lactating mothers on all days

(A) Free AA (µM)		(B) Total AA (mM)	(C) Various (µM)	
Ala	272 ± 115	6.5 ± 2.4	Creatinine	81.7 [70.8–86.2]
Thr	126 ± 46	6.8 ± 1.7	Sarcosine	2.43 [2.14–3.07]
Gly	235 ± 47	4.7 ± 1.2	ADMA	0.17 [0.14–0.19]
Val	59 ± 15	8.3 ± 2.2	pADMA	0.69 [0.43–1.03
Ser	188 ± 43	7.0 ± 1.6	pADMA/ADMA	4.29 [2.23–5.95]
Leu/Ile	125 ± 25	17.8 ± 4.4	Nitrate	42.6 [36.9–52.1]
Asp/Asn	139 ± 78	9.6 ± 2.6	Nitrite	29.1 [17.7–21.4]
Pro	83 ± 25	9.6 ± 2.6	Nitrate/nitrite	2.38 [1.72–2.71]
Met	170 ± 34	1.7 ± 0.3	MDA	0.62 [0.52–0.76]
Glu/Gln	1774 ± 465	20 ± 4.9		
Orn/Cit	5.7 ± 2.9	77 ± 18 (µM)		
Phe	31.3 ± 2.9	2.3 ± 0.6		
Tyr	43 ± 10	2.3 ± 0.6		
Lys	61 ± 23	5.9 ± 1.2		
Arg	27 ± 11	3.4 ± 1.0		
Trp	16.5 ± 3	55 ± 9 (µM)		

The concentrations of the free modified amino acids measured in the human breast milk samples are summarized in Table 2. Glycated amino acids, i.e., the advanced glycation end products (AGEs) including *N*^G^-carboxymethylarginine (CMA), were measured only in their free forms, because their proteinic precursors are unstable under the strong HCl-catalyzed hydrolysis conditions (110 °C, 20 h) (Iijima et al. 2000). Among the glycated amino acids, CMA was by far the most abundant PTM metabolite. To our knowledge, the present work is the first to report on the abundant occurrence of CMA in human breast milk. CMA has been first identified as a major AGE in collagen (Iijima et al. 2000). CMA can be formed chemically from glucose and methylglyoxal and was found in mice skin and in aorta of humans (Kinoshita et al. 2019). CMA may have a protective impact on intestinal cells of the neonates.

**Table 2:** Concentrations of the free PTM metabolites of Lys, Arg and Cys in the human breast milk samples of ten lactating women

Free PTM metabolite	Median [IQR] (nM)
D-5-Hydroxy-Lys	6 [5–7]
L-5-Hydroxy-Lys	11 [10–11]
*N*^ε^-Monomethyl-Lys (MML)	36 [17–53]
*N*^ε^-(2-Carboxymethyl)-Lys (CML)	16 [13–22]
*N*^ε^-(2-Carboxyethyl)-Lys (CEL)	5 [3–9]
*N*^ε^-(2-Furoylmethyl)-L-Lys (Furosine)	8 [6–8]
*N*^G^-Monomethyl-arginine (MMA)	38 [26–52]
*N*^G^-Carboxymethyl-arginine (CMA)	2452 [2019–3181]
*N*^G^-Carboxyethyl-arginine (CEA)	18 [11–24]
Dimethylamine (DMA)	4990 [4480–5850]
*S*-(2-Carboxymethyl)-Cys (CMC)	141 [76–236]
*S*-(2-Carboxyethyl)-Cys (CEC)	27 [10–28]
*S*-(2-Succinyl)-Cys (CSC)	32 [20–56]

**Fig. 1:**
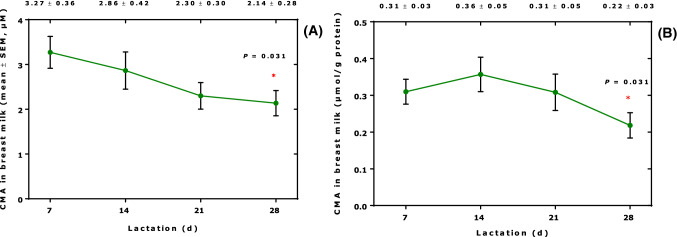
**A** Uncorrected and **B** protein-corrected *N*^G^-carboxymethyl-arginine (CMA) con-centration in human breast milk samples of 10 lactating women collected during the first month of lactation. Wilcoxon matched-pairs signed rank test was performed. Numbers on the top of both panels indicate the mean ± SEM concentrations of CMA measured on the respective lactation day (d)

Figure 1 shows that the concentration of free CMA in the human breast milk samples decreased with the duration of lactation. The CMA concentration corrected by the protein concentration in the milk samples was lower at lactation day 28 compared to lactation day 7.

Table 3 summarizes the statistically significant Spearman correlations found between the activities of SIRT1 and SIRT3 in the pellet (SIRT1pt and SIRT3pt) and in the aqueous (SIRT1aq and SIRT3aq) phase samples of the human breast milk. This Table also summarizes the Spearman correlations found between the SIRT1 and SIRT3 activity values and the concentrations of the listed metabolites. The analytes were assigned to PTMs, to few pathways and to individual amino acids.

**Table 3:** Spearman correlation coefficients (*r*) and statistical significance (*P*) between the SIRT1 and SIRT3 activity measured in the aqueous (aq) and pellets (pt) samples of the human breast milk and the indicated native and modified amino acids

Correlates	Positive correlations	Negative correlations
Sirtuins		
SIRT1pt − SIRT3pt	*r* = 0.581, *P* = 2.5E-4	
SIRT1pt − SIRT1aq	*r *= 0.635, *P* = 4.2E-5	
SIRT1pt − SIRT3aq	*r* = 0.376, *P* = 0.026	
SIRT3pt − SIRT1aq	*r *= 0.467, *P* = 0.0038	
SIRT3pt − SIRT3aq	*r* = 0.887, *P* = 1.3E-12	
SIRT1aq − SIRT3aq	*r* = 0.519, *P *= 0.0014	
Citrullination (CIT); arginine dimethylation (pADMA, DMA)		
SIRT1pt − CIT	*r* = 0.448, *P* = 0.0069	
SIRT3pt − CIT	*r* = 0.375, *P* = 0.026	
SIRT1aq − CIT	*r* = 0.533, *P* = 0.00097	
SIRT1pt − pADMA	*r* = 0.547, *P* = 0.0008	
SIRT1aq − pADMA	*r* = 0.389, *P* = 0.023	
SIRT3pt − DMA	*r* = *0.312, P* = *0.068*	
SIRT3aq − DMA	*r* = 0.358, *P* = 0.035	
Lysine hydroxylation; glycation; proline hydroxylation		
SIRT3pt − L-5-OH-Lys	*r* = 0.345, *P* = 0.043	
SIRT3aq − L-5-OH-Lys	*r* = 0.449, *P* = 0.0067	
SIRT1aq − CMA	*r* = 0.373, *P* = 0.027	
SIRT1pt − Furosine	*r* = *0.330, P* = *0.053*	
SIRT3aq − OH-Pro		*r* = − 0.361, *P* = 0.033
Arginase		
SIRT1pt − Orn/Cit		*r* = − 0.386, *P* = 0.022
SIRT3pt − Orn/Cit		*r* = − 0.403, *P* = 0.016
SIRT1aq − Orn/Cit		*r* = − 0.363, *P* = 0.033
SIRT3aq − Orn/Cit		*r* = − 0.349, *P* = 0.04
Agat (Arg:Gly amidinotransferase), GAMT (gaa methyltransferase) pathways		
SIRT1aq − GAA	*r* = 0.531, *P* = 0.001	
SIRT3aq − GAA	*r* = *0.327, P* = *0.055*	
SIRT1pt − *K*_harg_		*r* = − 0.382, *P* = 0.026
SIRT3pt − Creatinine	*r* = 0.537, *P* = 0.0009	
SIRT3aq − Creatinine	*r* = 0.630, *P* = 0.00005	
SIRT3pt − Met		*r* = − 0.527, *P* = 0.001
SIRT3aq − Met		*r* = − 0.486, *P* = 0.003
Nitric oxide pathway; oxidative stress		
SIRT3pt − Nitrate	*r* = 0.337, P = 0.048	
SIRT3aq − Nitrate	*r* = *0.321, P* = *0.063*	
SIRT3pt − Nitrite		*r* = − 0.524, *P* = 0.0012
SIRT3aq − Nitrite		*r* = − 0.431, *P* = 0.0097
SIRT3pt − MDA		*r* = − *0.323, P* = *0.058*
SIRT3aq − MDA		*r* = − *0.333, P* = *0.051*
Free Ala, Thr, Asp/Asn; total proteinogenic amino acids (pAA)		
SIRT1aq − Asp/Asn	*r* = 0.426, *P* = 0.011	
SIRT1aq − Thr	*r* = 0.374, *P* = 0.027	
SIRT3pt − Ala		*r* = − 0.366, *P* = 0.030

Data of all lactation days were considered. Terms in italics mean borderline correlation

The data presented in Table 3 were generated using the activity and the concentration values measured in all milk samples. The activity values of SIRT1 and SIRT3 correlated with each other. The highest correlations were observed for both sirtuins in the pellet and the corresponding aqueous phase: *r* = 0.635 (*P* = 4.2 × 10^−5^) for SIRT1pt/SIRT1aq, *r* = 0.887 (*P* = 1.3 × 10^−12^) for SIRT3pt/SIRT3aq. SIRT activities correlated positively with the citrullination degree (CIT), with ADMA in proteins (pADMA) and with free DMA, the major metabolite of free ADMA (Tsikas 2020).

SIRT3 and SIRT1 activity values correlated positively with the hydroxylation of Lys (L-5-hydroxy-Lys, L-5-OH-Lys; and D-5-hydroxy-Lys, D-5-OH-Lys) and with the glycation of Arg (i.e., CMA and furosine) (Table 3). Proline hydroxylation (5-hydroxy-Pro, OH-Pro) correlated inversely with the SIRT3aq activity. No correlations were observed for free *N*^ε^-monomethyllysine (MML), *N*^ε^-carboxymethyllysine (CML), *S*-carboxymethylcysteine (CMC) and *S*-carboxyethylcysteine (CEC).

SIRT1 and SIRT3 activity values correlated inversely with the arginase activity (measured as the sum of ornithine (Orn) and citrulline (Cit)) (Table 3). SIRT1 and SIRT3 activity values correlated positively with the concentration of guanidinoacetate (GAA), an arginine:glycine amidinotransferase (AGAT)-catalyzed metabolite of Arg and Gly, and with creatinine. SIRT1pt activity correlated inversely with the equilibrium constant of the AGAT-catalyzed formation of hArg, i.e., *K*_harg_. SIRT3 activity correlated inversely with the concentration of methionine (Met) in the human breast milk samples. SIRT3 activity correlated positively with nitrate, the major metabolite of nitric oxide (NO), but correlated inversely with nitrite, the minor metabolite of NO. Malondialdehyde (MDA), a biomarker of oxidative stress, notably of lipid peroxidation (Tsikas 2017), tended to correlate negatively with the SIRT3 activity. SIRT1aq activity correlated positively with the concentration of free aspartate/asparagine (Asp/Asn) and with the free threonine (Thr) concentration. SIRT3pt activity correlated inversely with the concentration of free alanine (Ala) in the breast milk samples. The concentration of the proteinic amino acids (pAA) obtained by HCl-catalyzed hydrolysis of milk proteins (110 °C, 20 h) did not correlate with either SIRT activity.

Previously, we found that the activity values of SIRT3aq and SIRT3pt in the breast milk were several times higher than those of SIRT1aq and SIRT1pt, and that they correlated inversely with the duration of lactation during the neonatal period (Hase et al. 2021). The highest SIRT1 and SIRT3 activity values were observed on lactation day 7 (Hase et al. 2021). We performed Spearman correlation analyses of the activity values of SIRT1aq, SIRT3aq, SIRT1pt and SIRT3pt among themselves, as well as with the concentrations of the analytes measured on each lactation day. The results of these analyzes are summarized in Table 4.

**Table 4:** Spearman correlation coefficients/statistical significance between the SIRT1 and SIRT3 activity values measured in the aqueous (aq) and pellets (pt) samples of the human breast milk and the concentrations of the indicated analytes measured in the milk samples on each lactation days (*n* = 9 for day 7; *n* = 10 for day 14; *n* = 9 for day 21; *n* = 7 for day 28)

Correlates	Day 7	Day 14	Day 21	Day 28
SIRT1pt − SIRT3pt	0.711/0.037	*0.636/0.054*	None	None
SIRT1pt − SIRT1aq	None	*0.632/0.056*	0.786/0.028	None
SIRT3pt − SIRT3aq	0.950/0.0004	0.782/0.011	0.929/0.002	0.857/0.024
SIRT1aq − SIRT3aq	None	None	*0.667/0.059*	None
SIRT1aq − CIT	None	*0.626/0.058*	None	None
SIRT1pt −pADMA	*0.553/0.065*	*0.515/0.089*	None	None
SIRT3pt −ADMA	None	None	None	− 0.927/0.007
SIRT3aq −ADMA	None	None	None	− 0.927/0.007
SIRT3pt −DMA	None	None	None	None
SIRT3aq −DMA	− *0.667/0.059*	0.709/0.027	None	None
SIRT3pt −D-5-OH-Lys	0.724/0.034	None	*0.621/0.082*	*0.655/0.128*
SIRT3aq − D-5-OH-Lys	*0.673/0.053*	None	*0.656/0.063*	None
SIRT3pt − OH-Pro	None	None	− 0.733/0.031	None
SIRT1aq − OH-Pro	None	None	− 0.683/0.050	− 0.919/0.006
SIRT1aq − GAA	None	0.592/0.036	0.717/0.037	None
SIRT3aq − GAA	None	None	0.800/0.014	None
SIRT1pt − hArg	*0.678/0.052*	None	None	None
SIRT3pt − hArg	0.717/0.037	None	None	− 0.929/0.007
SIRT1pt − Arg	*0.653/0.063*	None	None	None
SIRT1pt − *K*_harg_	None	None	None	− 0.762/0.037
SIRT3pt − Creatinine	None	0.661/0.044	0.905/0.005	*0.607/0.167*
SIRT3aq − Creatinine	None	*0.624/0.060*	0.833/0.015	0.821/0.034
SIRT3pt − Met	None	− 0.711/0.025	None	− 0.929/0.007
SIRT3aq − Met	None	− 0.729/0.020	None	− 0.786/0.048
SIRT3pt − Nitrite	− 0.721/0.023	− 0.717/0.024	None	None
SIRT3aq −Nitrite	− 0.758/0.015	− *0.571/0.089*	None	None
SIRT3pt −MDA	None	−* 0.624/0.060*	None	None
SIRT3aq − MDA	None	− 0.733/0.020	None	None
SIRT1aq−Asp/Asn	None	None	0.733/0.031	None

Numbers in italics indicate borderline significane

The activity values of SIRT3aq and SIRT3pt correlated closely with each other on all lactation days of the study (Table 4). SIRT3aq and SIRT3pt activity values correlated positively with the creatinine concentration on lactation days 14, 21 and 28, but not on day 7. In contrast, SIRT3aq and SIRT3pt activity values correlated inversely with nitrite on lactation days 7–14, but not on days 21–28. Among the PTM metabolites, 5-OH-Lys was found to correlate positively with SIRT3pt on lactation day 7, and borderline on lactation days 21 and 28. The type of correlation found on the individual lactation days was the same as for all lactation days taken together (Tables 3, 4).

## Discussion

**Fig. 2:**
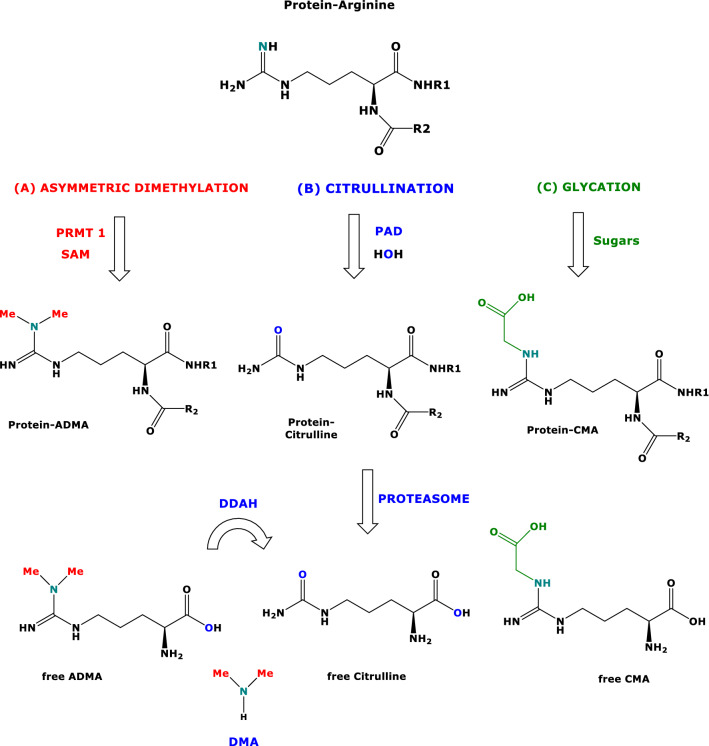
Simplified schematic of three major post-translational modifications of L-arginine residues in proteins. **A** Asymmetric dimethylation of L-arginine residues is catalyzed by protein-arginine methyl transferase 1 (PRMT1). **B** The conversion of L-arginine residues to L-citrulline residues is catalyzed by protein-arginine deiminase (PAD). **C** Glycation of L-arginine by sugars and their metabolites such as glyoxal yields carboxymethyl L-arginine residues. The proteasome releases the free ADMA, L-citrulline and carboxymethyl-L-arginine (CMA). Dimethylarginine dimethylaminohydrolase (DDAH) hydrolyzes free ADMA to free L-citrulline and dimethylamine (DMA). SAM, *S*-adenosylmethionine, cofactor of PRMT1. *Me* methyl

Asymmetric dimethylation, citrullination and glycation of Arg residues in proteins are major post-translational modifications (PTMs) (Fig. 2) (Tsikas 2021, 2022). Protein *N*-acetylation is one of the most frequent PTMs (Tsikas 2022) and plays a central role in regulating protein function. The function of sirtuins (SIRT) has been reported to be regulated by PTMs on the SIRT molecules themselves (Flick and Lüscher 2012; Kalous et al 2021). The metabolic functions of breast milk cells are little characterized thus far (Witkowska-Zimmy and Kaminska-El-Hassan 2017). The present study shows many associations of the activity of SIRT1 and SIRT3 with the concentration of major PTMs in human breast milk using samples of ten lactating women from a previous study (Hase et al 2021).

The strongest correlations were observed between SIRT1aq and SIRT1pt, and between SIRT3aq and SIRT3pt, when consider all lactation days together. SIRT3aq and SIRT3pt correlated with each other even on each separate lactation day. Correlations were found between SIRT1 or SIRT3 activity and different kinds of PTMs. The largest number of correlations were found when considering all lactation days together. The considerably lower number of significant correlations when considering separately the individual lactation days is likely to be due to the small number of lactating mothers (7–10 data sets per day compared to 35 data sets of the whole lactation period), the biological variability of all metabolites measured, and the decreasing activity values of SIRT activity. The present study suggests that SIRT1 and SIRT3 may play a role in the regulation of PTMs on other proteins and in other metabolic pathways of amino acids.

The occurrence of free ADMA in human breast milk has previously been reported (Tsukahara and Nagasaka 2010). To our knowledge, the present study is the first to report on the occurrence of citrullinated and asymmetrically dimethylated Arg residues in proteins present in human breast milk (Fig. 2). Additional PTMs found in human breast milk include the hydroxylation of Lys and Pro. With the sole exception of Pro hydroxylation, all correlations between SIRT1 or SIRT3 activity and PTMs were found to be positive.

The SIRT1 and SIRT3 activity in human mammary gland cells seems to block arginase activity and to influence the arginine:glycine amidinotransferase (AGAT) activity that leads to hArg and GAA. In turn, GAA is converted to the energy-related creatine by GAA *N*-methyltransferase (GAMT), which finally forms creatinine. Interestingly, SIRT3 activity was found to be differently associated with the NO metabolites nitrate (positive) and nitrite (negative). As nitrate and nitrite are metabolites of NO, this observation suggests that SIRT3 is involved both in the formation and oxidation of NO derived from the Arg/NO pathway. The borderline negative correlation of SIRT3 activity with MDA, a biomarker of lipid peroxidation (Tsikas 2017), suggests that SIRT3 activity may protect from lipid peroxidation in the lipid-rich breast milk.

In the present study, SIRT1 and SIRT3 activities were found not to correlate with any proteinogenic amino acid released by chemical proteolysis (6 M HCl) in the breast milk samples. The positive correlation of SIRT1 activity with concentration of free Asp/Asn and Thr, and the negative correlation of SIRT3 activity with the concentration of free Ala, a medium-term biomarker of lactate concentration (‘Cori cycle’), suggest that SIRT1 and SIRT3 may activate oxidative phosphorylation in mitochondria, thus reducing the need to produce energy via anaerobic glycolysis with lactate as the end-product. It is worth mentioning that free Thr in human breast milk was found to correlate negatively with the abundance of members of the class Gammaproteobacteria during early lactation, suggesting a potential protective role of Thr against members of the Enterobacteriaceae family in breast-fed infants (Riederer et al. 2022).

CMA was the most abundant AGE measured in human breast milk samples (Fig. 2). There is evidence that CMA is a representative AGE that serves as a useful index to reflect the oxidation and glycation of collagen (Kinoshita et al. 2019). The significance of free, peptidic and proteinic CMA in human breast milk and the expression of receptors for CMA and other AGEs (i.e., RAGEs) in human mammary gland cells remain to be investigated.

Breast milk is the gold standard for the nutrition of babies. Therefore, after elucidation of the physiological effects of sirtuins in breast milk, it may be appropriate to supplement formula feeding with sirtuins. This may even have an effect on health in later life according to the ‘concept of the first 1000 days’ (Maissan et al. 2021). Sirtuin-directed protein acetylation/deacetylation is involved in the regulation of host of diseases and metabolic syndrome and is regarded as a potential for targeted therapies (McGinnis et al. 2022). Protein acetylation is thought to direct feedback from metabolic mitochondrial pathways including beta-oxidation, the citric acid cycle, and the electron transport chain (McGinnis et al. 2022).

A potential limitation of our study is the relatively small number of lactating mothers. The correlations found between the activity of SIRT1 and SIRT3 and the concentrations of various native and modified amino acids including their metabolites such as creatinine, nitrite and nitrate, may not be causative. Although the correlations found between SIRT1 or SIRT3 and PTMs in our study do not necessarily mean that these sirtuins cause PTMs, their *N*-deacetylation activity is likely to modulate PTMs on Arg, Lys and Cys residues of certain proteins. In our study, we did not monitor the diet of the mothers during lactation. We cannot exclude that especially nitrate-rich diet may have contributed to the nitrate and nitrite content of the breast milk.

Due to the 1-month duration of the study, milk samples were frozen immediately. This is a generally used standard procedure for biological samples and should avoid decomposition of the milk components (Witkowska-Zimny and Kaminska-El-Hassan 2017). Due to the need of thawing, the milk samples for GC–MS analyses changes in the concentrations of the biomolecules cannot be fully excluded. Yet, based on our long-time experience with protein- and lipid-rich biological samples including plasma, no appreciable changes of the analytes in the milk samples are expected.

Human milk contains nutrients and several classes of low- and high-molecular-mass biomolecules including enzymes such as the secretory carbonic anhydrase (CA) VI (Karhumaa et al. 2001), catalase and superoxide dismutase (Gila-Díaz et al. 2020), hormones, growth factors, and microbiota (Selma-Royo 2021). All these factors contribute to the gut and immune system maturation of the neonates. The origin of SIRT1, SIRT3, CA VI and other constituents in breast human milk is unknown. Many of the biomolecules are implicated in infantile growth and development. It is possible that SIRT1, SIRT3 and CA VI are secreted by mammary gland cells. Many of the low-molecular-mass analytes we measured in the breast milk samples, including native and modified amino acids, nitrite, nitrate and creatinine, may also originate from the maternal blood and from other non-mammary gland cells, as well as from the nutrition of the lactating women and the saliva of mothers and neonates. Saliva may be of particular importance for the nitrate/nitrite/NO cycle (Kobayashi 2021). Our study design is not able to answer these questions.

Our previous study showed that breast milk contains active SIRT1 and SIRT3 (Hase et al. 2021). In the present study, we found correlations between the activity values of these sirtuins and the concentration of many biomolecules which can be assigned to different metabolic pathways. The nature of the enzymes that are involved in these pathways (e.g., AGAT, GAMT, NO synthase) and the PTM-modified peptides and proteins in the breast milk remains to be demonstrated.
